# HAP2/GCS1: Mounting evidence of our true biological EVE?

**DOI:** 10.1371/journal.pbio.3000007

**Published:** 2018-08-20

**Authors:** Theodore Clark

**Affiliations:** Department of Microbiology and Immunology, Cornell University, Ithaca, New York, United States of America

## Abstract

Sex has consequences—indeed, where would we be without it? Yet for all its importance, remarkably little is known about how sex evolved, why it has persisted, or even what mechanisms allow sperm–egg fusion to occur. Fortunately, answers to these questions are beginning to emerge with studies of hapless 2/generative cell specific1 (HAP2/GCS1), a molecular machine that promotes gamete fusion in organisms ranging from protists to flowering plants and insects. In studies by Fedry and colleagues, key structural features of the HAP2 protein are revealed for the first time, lending new insights into its mode of action and reinforcing its relationship to viral proteins that accomplish a similar task and may be intimately linked to the origins of cell–cell fusion events (including sexual reproduction) across evolutionary time.

## Abstract 2

Close structural relationships between proteins that mediate gamete fusion and class II viral fusogens support older theoretical arguments that sexual reproduction in eukaryotes arose with the horizontal transfer of a viral gene to a primordial cell early in the course of evolution.

## Introduction

Gametes, like all cells, are surrounded by biological membranes that partition them from each other and from the aqueous environment around them. Nevertheless, male and female gametes merge into a single cell at the onset of life, a process requiring that their respective membranes fuse. Gamete fusion is essential not only to our own survival but was likely a key hurdle that needed to be cleared for sex to have evolved in early eukaryotes. Nature solved that problem by finding a way to overcome the barriers that normally keep membranes apart, but how gametes accomplish this is only just beginning to become clear.

### The problem of gamete fusion

**Fig 1:**
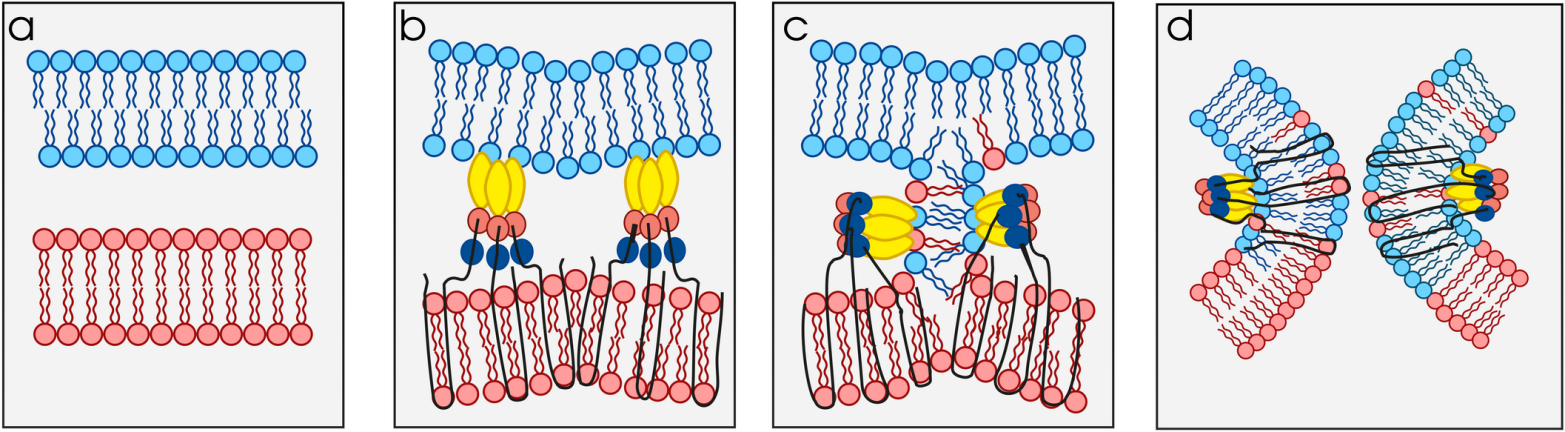
Membrane merger requires fusogens. a) Biological membranes are fluid structures comprised of lipid bilayers with polar head groups (blue and red circles) facing toward the aqueous environment on either side of the bilayer and hydrophobic acyl chains facing the interior. As biological membranes approach each other, the thin layer of water separating them acts as a repulsive force to keep them apart. b) The repulsive force of water is overcome by fusogens that span the two membranes and bring them together. This panel shows class II viral fusogens, which have three characteristic domains denoted in dark blue (domain III), red (domain I), and yellow (domain II). Class II fusogens bridge the membrane of the virus with the membrane of an endocytic vacuole in which the virus is taken up. The fusogen is linked to the virus envelope via a membrane-spanning anchor connected to domain III (bottom) and interacts with the membranes of endocytic vacuoles (top) via hydrophobic fusion loops at the distal end of domain II. Individual class II monomers assemble into trimers during the fusion process. c) Class II fusogens fold back on themselves to bend apposed membranes and drive fusion of the outer leaflets first to form a hemifusion intermediate. This conformational change is generally triggered by the low-pH environment of the endosomes. d) As the fusogens continue to fold back, the hydrophobic membrane-spanning anchors and fusion loops on either side of the proteins are thought to destabilize the hemifusion intermediate and allow fusion of the inner leaflets, opening a pore that connects the interior of the virus with the host cell cytosol.

Biological membranes are comprised of phospholipid bilayers in which sterols, proteins, and other substances are embedded. For cells to fuse, they must first approach each other, but as their plasma membranes get very close (approximately 10 nm), the thin layer of water separating them acts as a strong repulsive force that prevents the two bilayers from merging into a single, continuous sheet. Indeed, the closer membranes get to one another the stronger the repulsive force of water becomes [1,2]. Despite this, biological membranes can fuse, and there are numerous examples in which somatic cells, cells and viruses, and intracellular organelles join together via membrane fusion (Box 1). Almost invariably, these processes rely on “fusogens,” a remarkable set of proteins that can store energy and act as catalysts to overcome the hydration force that repels membranes when they get too close [1–4] (Fig 1).

**Box 1. Examples of membrane fusion.**

A variety of cell types are capable of fusion. Well-studied examples include myoblasts, which give rise to muscle tissue in vertebrate and invertebrate animals and cytotrophoblasts, a type of epithelial cell that forms the syncytiotrophoblast of the placenta of mammals. In each case, cell–cell fusion results in the formation of syncytia or multinucleated cells. Both processes require that fusogens catalyze the merger of adjoining cells. In the case of vertebrate muscle, two recently identified proteins, Myomaker and Myomerger–Minion, act in concert to mediate myoblast fusion, although the underlying basis of this system is still under study [5]. In the case of the syncytiotrophoblast, the fusion proteins are referred to as “syncytins,” which are encoded by genes for retroviral fusogens that spread to mammalian genomes via horizontal gene transfer millions of years ago [6].

From a mechanistic standpoint, perhaps the best-understood fusion proteins are those involved in vesicle-mediated protein sorting and those that control infection of cells by enveloped viruses. In the first case, merger of transport vesicles (e.g., late endosomes) with either target organelles (e.g., lysosomes) or with the plasma membrane (as in the case of peptide hormone or neurotransmitter release) involves the formation of so-called soluble NSF (N-ethylmaleimide sensitive-factor) attachment protein receptor (SNARE) complexes between the helical regions of proteins anchored to vesicles and target membranes, respectively. Interactions between the SNARE proteins followed by “zippering-up” of the helical regions brings apposed membranes together. In the case of enveloped viruses (which are themselves surrounded by membranes), there are three characterized classes of fusogens (designated class I, II, and III) that differ in their overall structures. In all cases, they bring the virus envelope together with either the plasma membrane or the membrane of an endocytic vesicle in which the virus has been taken up. Fusion of apposed membranes leads to the release of viral nucleic acids into the host cell cytosol, the first step in virus replication [2].

There are a number of basic concepts that govern membrane fusion reactions. First, regardless of the system involved, fusion is almost always targeted. That is, a given cell or membrane system will fuse only with a specific cell or target membrane. In general, sperm from one species will only fuse with eggs of the same species, the fidelity of these reactions defining species boundaries. Related to this, fusion is often preceded by recognition and adherence of the membranes that are destined to fuse. These initial steps are generally controlled by receptor–ligand interactions that determine the specificity of the fusion reaction. Sometimes the fusogen binds a protein receptor on a target cell but not always. Finally, after recognition and adherence are achieved, fusogens drive membrane merger in a step-wise fashion, beginning with the outer leaflets of the apposed lipid bilayers coming together to form what is referred to as a hemifusion intermediate (or stalk), followed by destabilization and merger of the inner leaflets, leading to the opening of a pore that effectively joins the compartments separated by the fusing membranes (Fig 1). The energy that drives these reactions comes from changes in the three-dimensional conformations of the fusogens themselves.

Given their widespread involvement in membrane fusion, it has generally been assumed that fusogens are also required for fertilization; however, in the case of vertebrates, this is still not known. Recent studies indicate that the sperm protein Izumo1 and its binding partner on eggs, Juno, are necessary for attachment and fusion of gametes in mammals, but there is little evidence that these or other proteins linked to fertilization in mammals are bona fide fusogens [7]. A role for fusogens in sexual reproduction in nonvertebrate species is nevertheless clear cut based on studies of the gamete-specific protein HAP2/GCS1.

### HAP2/GCS1: An ancient gamete fusogen

*HAP2* and its orthologous gene sequence *GCS1* were identified in different plant species by Mark Johnson and colleagues in the United States and Toshiyuri Mori’s group in Japan in the mid-2000s [8–10]. In the first instance, mutant lines of *Arabidopsis thaliana* that failed to produce fertile male gametes were identified and assigned the prefix “hap” (for “hapless”). A mutation in one such line (*hap2*) was traced to a gene that appeared to control pollen tube guidance and fertilization [8,9]. In parallel, Mori’s group discovered a messenger RNA transcript and corresponding gene (termed *GCS1*) in the trumpet lily *Lilium longiflorum*, whose expression was highly up-regulated during the late stages of development of generative cells, the precursors of sperm [10]. The *HAP2* and *GCS1* genes encoded very similar proteins with a single membrane-spanning domain, an extracellular domain of approximately 600 amino acids and a relatively short cytoplasmic tail. As expected, the HAP2/GCS1 protein localized to the surface of sperm cells and was shown to be necessary for fertilization of the egg in *Arabidopisis* [9,10].

Although *HAP2/GCS1* was originally described in plants, orthologous genes were quickly identified in single-celled protists (including *Plasmodium falciparum*, the causative agent of malaria), unicellular and colonial algae (*Chlamyodomonas reinhardii* and *Gonium pectorale*), as well as invertebrate animals (*Hydra* and honeybees) [11–13]. Indeed, the *HAP2/GCS1* gene has now been found in representative species of almost all major branches of the eukaryotic tree of life, including some of the oldest extant lineages [14]. Such phylogenomic studies date the emergence of *HAP2/GCS1* to the last eukaryotic common ancestor (LECA) at least 1 billion years ago.

The requirement for *HAP2/GCS1* for fertilization in *Arabidopsis* was clearly suggestive of a possible role in gamete fusion and prompted a rash of additional studies in other systems. Working with the green algae *C*. *reinhardtii*, William Snell and colleagues showed that expression of the *HAP2* gene in the minus mating type (the equivalent of male gametes in this species) was absolutely required for gamete fusion and that the HAP2 protein localized to the tip of a finger-like projection on these cells precisely where fusion with the plus (female) gamete begins [15]. Along the same lines, studies of the pond water ciliate *Tetrahymena thermophila* showed that HAP2 was required for the fusion of diverse mating types and localized to a specific structure, the conjugation junction, where gametes fuse, exchange haploid pronuclei, and consummate sex [16].

While evidence was mounting that HAP2/GCS1 orthologs might indeed be fusogens, their primary amino acid sequences were unlike other known proteins, making it difficult to infer their true function. However, what came next was quite unexpected. Using computer algorithms that allow one to model protein structures, three separate groups predicted that the extracellular regions of HAP2/GCS1 from *Chlamydomonas*, *Tetrahymena*, and *Arabidopsis*, respectively, would adopt structures that closely mimic class II fusogens from dengue, Zika, and related animal viruses [17–19]. At the same time, Felix Rey’s group at the Pasteur Institute in France determined an actual structure for the *Chlamydomonas* HAP2/GCS1 extracellular domain via X-ray crystallography, offering proof that the gamete protein and viral fusogens share the same overall tripartite structure rich in β-strands [17].

Fedry and colleagues now extend these observations with X-ray crystal structures of the extracellular domains of HAP2/GCS1 from *A*. *thaliana* and the protozoan parasite *Trypanosoma cruzi* [20]. While the earlier determination of the *Chlamydomonas* HAP2/GCS1 crystal structure was a towering achievement, this new data sheds light on regions of the protein (the fusion loops) that are critical to activity but were missing in the previous study. All class II viral fusogens are anchored at one end to the virus envelope via hydrophobic transmembrane domains. However, in order for class II fusogens to work, they must bridge viral and cellular membranes, which requires that their distal ends interact with target cell membranes as well (Fig 1). This interaction involves so-called fusion loops that are themselves hydrophobic and insert into the bilayers of the apposed cellular membranes.

Structural studies of *Chlamydomonas* HAP2/GCS1 had failed to capture the region predicted to form the fusion loop(s), making it difficult to say whether this region actually serves as a membrane anchor. Indeed, the absence of structure in this region left open the possibility that HAP2/GCS1 might use an alternative strategy to drive membrane fusion akin to what had previously been proposed for the developmental fusogen epithelial fusion failure-1 (EFF-1) that controls cell–cell fusion in developing tissues of nematode worms. Like HAP2/GCS1, EFF-1 is structurally related to class II viral fusogens but lacks an obvious fusion loop [21]. This led to the idea that EFF-1 may interact with itself or with other proteins on adjoining cells to bring membranes together without the need for the distal end of the fusogen to insert into a lipid bilayer [21]. A similar argument has been made for HAP2/GCS1 based on evidence that membrane fusion may be more efficient when the protein is expressed on the plasma membranes of two apposed cells [19]. Nevertheless, the present work of Fedry and colleagues captures the structures of the fusion loops of both the *A*. *thaliana* and *T*. *cruzi* proteins and demonstrates unequivocally that loop structures can interact with phospholipid bilayers, thus obviating the need for protein–protein interactions across membrane surfaces for HAP2/GCS1-mediated fusion to occur.

Comparisons of HAP2/GCS1 crystal structures from *Arabidopsis*, *T*. *cruzi*, and *C*. *reinhardtii* indicate that all three proteins share the same overall design, with several regions being highly conserved. Surprisingly, however, the fusion loop structures on the plant and parasite proteins are quite different from each other, one having three short loops comprising a rather flat membrane interacting surface (*T*. *cruzi*) and the other a single prominent loop with an amphipathic helix jutting out and well positioned to interact with the apposed membrane (*A*. *thaliana*). Presumably, these differences evolved to accommodate differences in the composition of the membranes with which they interact.

In short, the work of Fedry and colleagues defines several conserved structural motifs along with interesting differences in the membrane-interacting domains of HAP2/GCS1 proteins from two widely diverged eukaryotic species. The conserved motifs may facilitate identification of related proteins in species that lack obvious HAP2/GCS1 orthologs based on primary sequence comparisons alone, while differences in the fusion loop motifs raise new questions about the importance of the lipid environment in shaping HAP2/GCS1 structure.

With all that we now know, there is still much to be learned about HAP2/GCS1. In terms of structure–activity relationships, X-ray crystal structures are snapshots in time capturing proteins in specific conformations—postfusion trimers in the current work on HAP2/GCS1. While informative, structures of prefusion intermediates would also be useful in telling us how changes in protein conformation actually drive membrane fusion. Additionally, we can only assume that conformational changes in HAP2/GCS1 are tightly regulated in time and space to prevent misdirected fusion events. This is clearly the case for viral fusogens, which undergo conformational rearrangements in response to specific cues, but what the triggers are for HAP2/GCS1 refolding remains to be determined. Likewise, HAP2/GCS1 localizes to specific regions of the plasma membranes of male gametes. Presumably, accessory proteins or chaperones guide the fusogen to these locations, but the identity of those proteins and whether they also play a role in triggering conformational change is still unknown.

From an evolutionary standpoint, questions surrounding the origins of HAP2/GCS1 are equally interesting. First and foremost, given its ancient ancestry and the strict requirement for cell–cell fusion in sexual reproduction in almost all species, emergence of the HAP2/GCS1 gene may have been instrumental in the evolution of eukaryotic sex. The possible link between viral fusogens and HAP2/GCS1 makes that argument all the more intriguing. Viruses fuse with cells in order to reproduce, but, in doing so, they often exchange genetic information with their hosts, leaving behind traces of DNA known collectively as endogenous viral elements (EVEs) [22]. Indeed, much of our own DNA came from viruses, and some of that has been repurposed for our own use. A notable example in this case is genes that specify syncytins, a family of fusogens that regulate development of the syncytiotrophoblast, a critical layer of the placenta of mammals. Remarkably, the DNA encoding these proteins are remnants of genes for class I fusogens from retroviruses that invaded animal genomes on multiple occasions between 30–80 million years ago [6].

**Fig 2:**
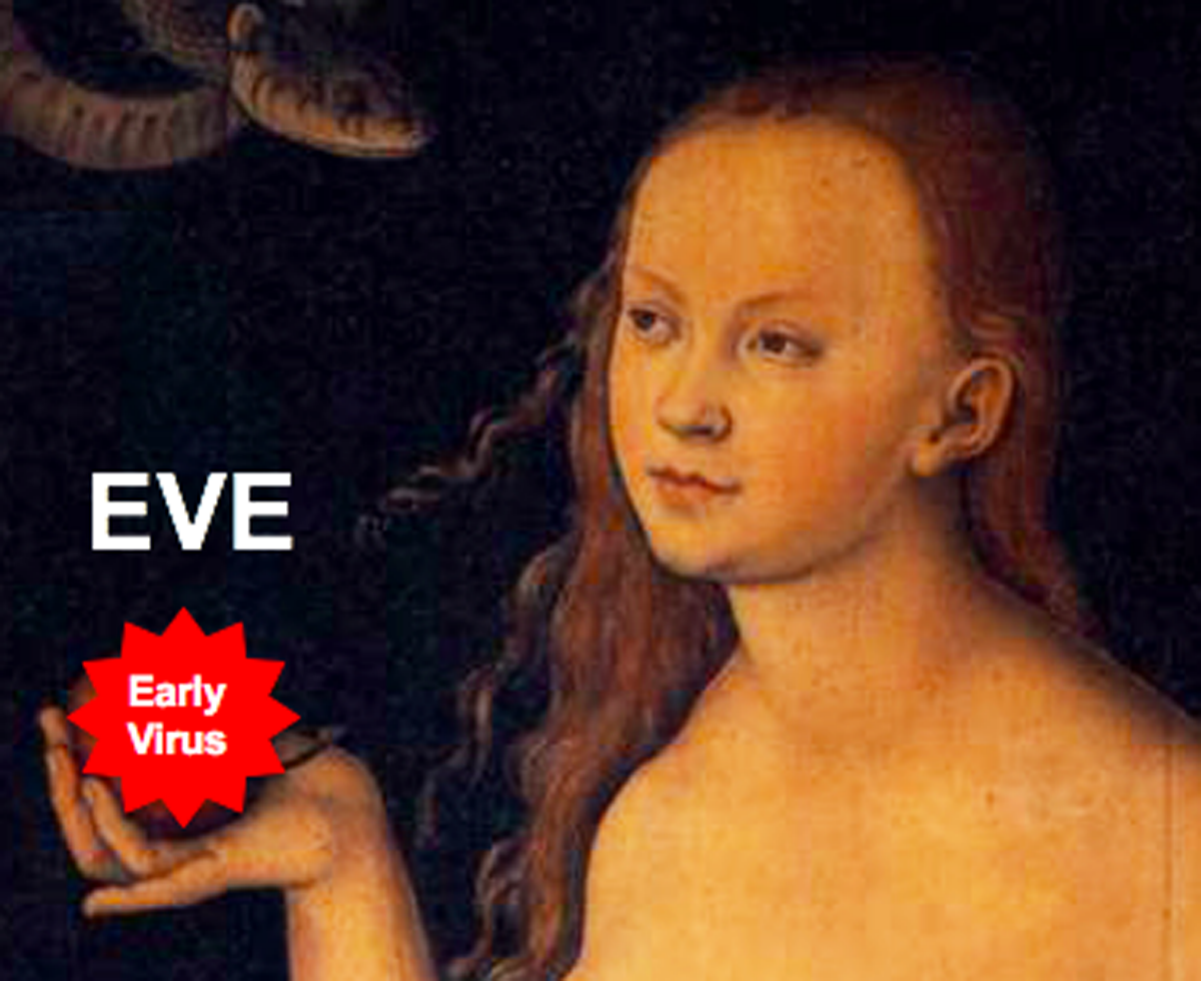
Did an EVE spawn the eukaryotic sexual revolution? Studies of HAP2/GCS1 suggest that a key step in sexual reproduction, namely gamete fusion, may have arisen with the horizontal transfer of a viral gene to a primordial eukaryotic cell early in the course of evolution. Absent that step, it is unclear whether the diversity of life as we know it today would ever have evolved. Alternatively, HAP2/GCS1 may have evolved through natural selection in eukaryotes and been captured by viruses at some point in evolution. Although we can trace the *HAP2/GCS1* gene to the last eukaryotic common ancestor, structurally related proteins may date to archaea (D. Moi, X. Li, H. Romero, P. Aguilar and B. Podbilewicz, personal communication). EVE, endogenous virus element; HAP2/GCS1, hapless 2/generative cell specific1.

Along the same lines, recent evidence has suggested that genes for class II viral fusogens have been captured independently from host cell genomes by unrelated viruses at different times in the past [23]. This would imply that cellular genes for class II-like proteins are present in eukaryotic genomes, an obvious example being *HAP2/GCS1*. Based on similarities in their protein-folding patterns and the low probability that this came about through convergent evolution, Rey and others have argued that class II viral fusogens and HAP2/GCS1 evolved from a common ancestor [17–19]. If true, however, did the ancestral gene come from a virus, or did viruses capture it from an early eukaryote, perhaps replacing an older, less efficient fusogen [24]? While we may never know, the question itself takes us back to the origin of eukaryotic sex and the revelatory possibility that sexual reproduction and the myriad of life forms that exist on earth were all made possible by a snippet of DNA from a virus (Fig 2). For the interested reader, the idea that the evolution of sex was driven by a parasitic viral DNA element was proposed >30 years ago by Donal Hickey on strictly theoretical grounds [25,26].

Finally, while HAP2/GCS1 clearly plays a vital role in sexual reproduction in many eukaryotic lineages, it is missing (or has not yet been found) in humans and other vertebrates. While it is entirely possible that we utilize a different mechanism for sperm–egg fusion, the work of Fedry and colleagues points to conserved structural elements in HAP2/GCS1 that may be instrumental in helping us find related proteins in species such as our own.

## Abbreviations

EFF-1: epithelial fusion failure-1
EVE: endogenous viral element
HAP2/GCS1: hapless 2/generative cell specific1
LECA: last eukaryotic common ancestor
NSF: N-ethylmaleimide sensitive-factor
SNARE: soluble NSF attachment protein receptor
